# Prediction of miRNA-disease associations based on PCA and cascade forest

**DOI:** 10.1186/s12859-024-05999-w

**Published:** 2024-12-19

**Authors:** Chuanlei Zhang, Yubo Li, Yinglun Dong, Wei Chen, Changqing Yu

**Affiliations:** 1 Artificial Intelligence, Tianjin University of Science and Technology, Tianjin, 300457 China; 2 Computer Science, China University of Mining and Technology, Xuzhou, 221116 China; 3 Electronic Information, Xijing University, Xi’an, 710123 China

**Keywords:** miRNA-disease Association, PCA, Cascade forest, Ensemble learning

## Abstract

**Background:**

As a key non-coding RNA molecule, miRNA profoundly affects gene expression regulation and connects to the pathological processes of several kinds of human diseases. However, conventional experimental methods for validating miRNA-disease associations are laborious. Consequently, the development of efficient and reliable computational prediction models is crucial for the identification and validation of these associations.

**Results:**

In this research, we developed the PCACFMDA method to predict the potential associations between miRNAs and diseases. To construct a multidimensional feature matrix, we consider the fusion similarities of miRNA and disease and miRNA-disease pairs. We then use principal component analysis(PCA) to reduce data complexity and extract low-dimensional features. Subsequently, a tuned cascade forest is used to mine the features and output prediction scores deeply. The results of the 5-fold cross-validation using the HMDD v2.0 database indicate that the PCACFMDA algorithm achieved an AUC of 98.56%. Additionally, we perform case studies on breast, esophageal and lung neoplasms. The findings revealed that the top 50 miRNAs most strongly linked to each disease have been validated.

**Conclusions:**

Based on PCA and optimized cascade forests, we propose the PCACFMDA model for predicting undiscovered miRNA-disease associations. The experimental results demonstrate superior prediction performance and commendable stability. Consequently, the PCACFMDA is a potent instrument for in-depth exploration of miRNA-disease associations.

## Introduction

MiRNAs typically consist of approximately 20–24 nucleotides and regulate the function of target messenger RNA (mRNA) at the post-transcriptional stage by precisely matching the 3’ non-coding region of the target mRNA [1]. In the realm of human RNAs, aside from mRNA, which is responsible for protein synthesis, there are various non-coding RNAs including snRNAs [2], circRNAs [3, 4] and lncRNAs [5, 6]. Although these non-coding RNAs do not encode proteins, their importance in regulating biological processes such as embryogenesis, stem cell homeostasis, cellular differentiation, metabolic regulation, signaling pathways, and immune response has been widely demonstrated [7]. Current research has revealed immediate and complex connections between miRNAs and diseases. The abnormalities in their expression levels are often closely associated with the processes underlying the occurrence of various complicated diseases [8]. Therefore, revealing the functional properties of unknown miRNAs and their specific roles in the disease process not only offers novel insights for therapeutic target identification and drug development but also promotes the development of miRNA-based biomarkers. These biomarkers can greatly facilitate the diagnostic accuracy of diseases and bring advances in clinical treatment strategies.

Exploration of miRNA-disease association (MDA) has benefited much from conventional experimental methods like polymerase chain reaction (PCR) [9] and reverse transcription polymerase chain reaction (RT-PCR) [10]. The cancer [11] is usually diagnosed at a late stage, which severely limits the effectiveness of therapeutic interventions. Therefore, it is essential to construct accurate miRNA-disease prediction models. These models can significantly improve the efficiency and speed of laboratory validation. Furthermore, multidisciplinary research has demonstrated that the molecular interaction networks in organisms are intricate and complex, including protein-disease, gene-disease, microbe-disease, and non-coding RNA-protein interactions [12], which provide novel perspectives for predicting MDA. For example, Deng et al. [13] successfully identified several key genes closely related to cervical carcinogenesis using various bioinformatics tools. Zhao et al. [14] innovatively employed gene expression profiling instead of miRNA expression data or gene-miRNA pairing information. In addition, Yi et al. [15] constructed a highly integrated heterogeneous molecular association network, offering a valuable structure for comprehending the synergistic effects of these molecules in disease progression.

In bioinformatics research, many methods based on mathematical statistical analysis have been developed. These methods not only aid in addressing complex problems in bioinformatics but also promote the interdisciplinary exchange between biology and statistics. For instance, the CMFMDA model developed by Shen et al. [16] leverages efficient mathematical algorithms, enhancing prediction accuracy while maintaining computational speed. Additionally, Gao et al. [17] implemented the NPCMF model on the framework of traditional matrix decomposition. The model uniquely incorporates relevant considerations of emerging miRNA and disease entities with their neighboring node information. It cleverly combines the concept of nearest-neighbor contour analysis (NP) [18], which greatly optimizes the prediction efficacy. Furthermore, Yao et al. combined the negative instance inference strategy with the low-rank matrix complementation approach [19] to deepen the inference capability for unassociated miRNA-disease pairs. They comprehensively consider the complexity of heterogeneous network environments and alleviate the problem of an insufficient number of negative samples. Ha et al. proposed the IMIPMF model [20] for unknown miRNA-disease associations, inspired by Probabilistic Matrix Factorization (PMF) in recommendation systems. Subsequently, Ha proposed the MDMF framework by adding disease similarity information to the matrix factorization [21], after which Ha also proposed the SMAP model [22], which integrates comprehensive information on miRNA and disease similarity into the matrix factorization framework. Additionally, Ha et al. [23] combined the linear function of Generalized Matrix Factorization (GMF) and the nonlinear capability of Multilayer Perceptron (MLP) to improve the prediction accuracy. Finally, Ha proposed a matrix decomposition method based on lncRNA expression profiles (EMFLDA) [24] for identifying lncRNA-disease associations by learning the latent space shared by lncRNAs and diseases and minimizing the difference between the original matrix values and the products of rows and columns of the latent space. The EDTMDA [25] ensemble learning framework proposed by Chen et al. cleverly incorporates PCA into each basic learning module to improve the model’s efficiency. Yu et al. [26] used Tensor Robust Principal Component Analysis (TRPCA) to investigate the MDA. In contrast to previous approaches using binary associations, ternary associations of $\langle miRNA, disease, type \rangle$ were used to characterize complex relationships. Rajapandy et al. [27] transformed MDA data into a low-dimensional representation space via PCA. Furthermore, Liu et al. creatively combined the attention mechanism of the Graph Convolutional Neural Network (GCN) with PCA in the GCNPCA [28] method to enhance the explanatory power.

Machine learning methods excel in identifying patterns and regularities from large datasets. For example, Jiang et al. [29] demonstrated the effective application of SVM in predicting the MDA. Chen et al. [30] proposed the DRMDA model, which combines SVM and autoencoder (AE) for association prediction, demonstrating the potential of SVM in complex bioinformatics prediction tasks. However, a major limitation of this supervised learning algorithm is its heavy reliance on the completeness of positive and negative samples. It requires sufficient known associated and unassociated data as a training basis, which may pose a major challenge in practical applications. The RKNNMDA model innovatively combined the KNN and SVM ranking methods [31] to enhance the performance of MDA prediction. The MSCHLMDA [32] model merges KNN and K-means to form a two-layer hypergraph structure, showcasing how algorithm combinations can enhance prediction model performance. In the development of AMNDA [33], Chen et al. cleverly applied K-means clustering to screen unlabeled miRNA-disease pairs. They enhanced the stability and noise resistance of the model by evenly sampling negative examples from different clusters. In metric learning, the similarity between objects is transformed into the corresponding distance metric, which overcomes the problem of not conforming to the triangular inequality prevalent in matrix decomposition-based methods. The MLMD model proposed by Ha et al. [34], constructs miRNA-disease bipartite graphs and uses distance metric learning to infer miRNA-disease associations. The proposal of the RFMDA model [35] marked the initial success of RF. IRFMDA model [36] further optimized the screening and utilization of features by introducing the variable radio frequency score. The MDA-CF model [37] used an autoencoder for feature dimensionality reduction, followed by the application of cascaded forests on the optimized feature space, which embodies the effective integration of deep learning with traditional machine learning algorithms. The DFELMDA model [38] and the CFSAEMDA method [39] then further deepened this combination by realizing an advanced integration of deep and integrated learning of feature representations and improving prediction accuracy through deep random forests and stacked autoencoder, respectively. Gradient boosting tree algorithms, such as XGBoost, LightGBM and CatBoost, show significant advantages in handling large-scale datasets and improving prediction accuracy. The clinical decision support system constructed using these algorithms in a study by Kim et al. [40] not only pushed forward the development of healthcare AI but also offered new perspectives on MDA prediction. Similarly, the KS-CMI model [41] combined denoising autoencoder and CatBoost, enhancing feature representation and bolstering prediction robustness through balance theory. Furthermore, Zhao et al. [42] explored the application of adaptive enhancement techniques in complex metric spaces. Then the ABPUSVM model proposed by Zhong et al. [43] creatively combined the Positive-Unlabeled learning strategy and SVM to address the sample imbalance in the MDA prediction problem.

Deep learning approaches can analyze massive amounts of high-dimensional biological data, allowing for more accurate and efficient bioinformatics analysis and prediction. For example, Chen et al. [44] pre-trained a restricted Boltzmann machine using all miRNA-disease associations to reduce the impact of limited known associations on prediction accuracy. They then fine-tuned a Deep Belief Network (DBN) with the same number of positive and negative samples and obtained the prediction results. Ji et al. [45] implemented a semi-supervised learning strategy that combines the expression features and applies Variational Autoencoder (VAE) to the existing MDA to reveal unknown correlations. The GAEMDA algorithm [46] employs a Graph Autoencoder (GAE) to construct miRNA-disease features of low-dimensional embeddings and employs a bilinear decoding mechanism to parse the association between them. On the other hand, Wang et al. [47] processed these features with the help of stacked GAE, transformed them into low-dimensional representations, and finally predicted miRNA-disease interactions using MLP. In addition, VGAE-MDA [48] extracts features from the composite network of miRNAs and diseases and quantifies the strength of their association by Variational Graph Autoencoder (VGAE). This framework optimizes MDA prediction by integrating the prediction scores of different subnetworks, effectively mitigating the noise problem introduced by random negative case selection, and fuses the strengths of GCN and VAE. The MSCNE model [49] innovatively integrated Convolutional Neural Network with AE to create a multi-level feature extraction subnetwork for the final association prediction. The MDA-GCNFTG method [50] relied on the greedy strategy of GCN and graph sampling to solve the common problem of proliferation of the number of neighboring nodes in GCN. Further, the LAGCN model [51] strengthens the learning efficacy of GCN through multilayer convolution and attention-guided embedded representation learning. The NSAMDA model [52] marked a turning point by introducing the attention mechanism into spatial-domain-based graph neural networks, which achieves effective aggregation of node features. Compared with traditional approaches that rely on complex matrix operations, this model only needs to consider first-order neighborhood information. Li et al. [53] then implemented attention learning at the node and semantic levels by constructing a hierarchical graph attention network model as a means to assess the significance of different meta-paths and used a bilinear decoder to recover potential links, demonstrating a strong feature differentiation capability. Finally, the AMHMDA method proposed by Ning et al. [54] fused multi-view networks with hypergraph learning and used the attention mechanism to integrate multi-view outputs from GCN. Specifically, the principal contributions of this research are enumerated as follows:

- We innovatively fuse PCA and Cascade Forest (CF) to predict MDA, which is enhanced by a two-stage strategy. Firstly, the miRNA-miRNA similarity information, disease-disease similarity information and validated miRNA-disease association information are integrated to obtain a comprehensive feature representation of miRNAs and diseases. Then PCA is applied to obtain the key information of these features. Finally, an optimized cascade forest is used for prediction and get the final result.
- We implement 5-fold and 10-fold cross-validation methods on the HMDD v2.0 database. The PCACFMDA model achieves 98.56% and 98.58% AUC values. In addition, experimental comparisons of the reduced dimensionality data using multiple classification algorithms are performed. A comprehensive evaluation of our model with other relevant and similar predictive models is also performed.
- To further confirm the practical value of the PCACFMDA model, three common diseases are selected as case studies for a comprehensive evaluation. The possible MDAs calculated by the model have been confirmed by independent experimental studies, confirming the approach’s great accuracy and reliability in discovering true biological connections.

## Methods

### Dataset

HMDD v2.0 contains 495 miRNAs, 383 diseases, and 5430 experimentally validated associations, as well as 184,155 unvalidated potential associations [55]. To systematically represent the MDA, we constructed an adjacency matrix A(i,j) with 495 rows and 383 columns. The corresponding elements A(i,j) of matrix A are assigned a value of 1 if miRNA i is associated with disease j, and 0 otherwise. In evaluating the model’s performance, we employed a balanced sampling strategy, i.e., using the 5430 confirmed associations as the positive sample set. At the same time, an equal number of 5430 unlabeled associations were randomly selected from the 184,155 unlabeled associations as the negative sample set, ensuring a balanced sample set for a more precise assessment of model’s effectiveness. Table 1 demonstrates the detailed sample distribution.

**Table 1:** Dataset sample

Dataset	Known MDA	Unknown MDA
Original	5430	184,155
Balanced	5430	5430

Numerous human health problems such as cancer, neurological diseases, cardiovascular diseases, and metabolic abnormalities are closely related to miRNA regulatory dysfunction. Considering that miRNA involved in similar pathological processes may have similar biological functions, we can obtain raw miRNA functional similarity data concerning previous studies [56]. The MFS ($m_i$, $m_j$) in this study indicates the functional similarity of two miRNAs.

To obtain disease associations, we used a Directed Acyclic Graph (DAG) [57] to characterize the Medical Subject Headings (MeSH), which is adept at describing complex causal or subordinate links between elements. In quantifying the semantic similarity (DSS) between diseases, two core algorithmic strategies are adopted: the first algorithm considers that two diseases with a higher number of shared parent nodes in the DAG are more similar to each other; the other algorithm emphasizes that diseases at different levels have hierarchical differences in semantic importance. The first approach does not take into account the frequency of recurrence of diseases in the DAG structure. The second approach compensates for this by assigning different weights to diseases at the same level. Combining these two considerations, we arrived at a more comprehensive semantic similarity score of diseases by calculating their average value, which serves as the baseline result of the study. The DSS ($d_i$, $d_j$) in this study indicates the semantic similarity of the two diseases.

Disease characteristics often show associations with functionally similar miRNAs, whereas functionally unrelated miRNAs are typically linked to distinct disease profiles [58]. The Gaussian Interaction Profile (GIP) kernel similarity [59] approach is employed to quantify the level of functional association among miRNAs. The calculation formulas are as follows:

1 $$\begin{aligned} GMS(m_i,m_j)= & \exp (-\gamma _m\Vert IP(m_i)-IP(m_j)\Vert ^2) \end{aligned}$$

2 $$\begin{aligned} \gamma _m= & \frac{\gamma _m^{\prime }}{\frac{1}{n_m}\sum _{i=1}^{n_m}\Vert IP(m_i)\Vert ^2} \end{aligned}$$

where $\gamma _m$ controls the kernel bandwidth and $n_m$ is the number of miRNAs, $\gamma _m^{\prime }$ equal to 1. Similarly, we can calculate the GIP kernel similarity between two diseases using the following formula:

3 $$\begin{aligned} GDS(d_i,d_j)= & \exp (-\gamma _d\Vert IP(d_i)-IP(d_j)\Vert ^2) \end{aligned}$$

4 $$\begin{aligned} \gamma _d= & \frac{\gamma _d^{\prime }}{\frac{1}{n_d}\sum _{i=1}^{n_d}\Vert IP(d_i)\Vert ^2} \end{aligned}$$

Some miRNAs lack functional similarity, and certain diseases may not exhibit semantic similarity. Therefore, for miRNAs, we integrated information from both MFS and GFS. When the MFS for any pair of miRNAs is non-zero, we use the average of MFS and GFS to represent the fusion similarity. If the MFS is zero, it indicates that they are not directly functionally related, in which case we rely solely on the GFS to estimate their similarity. Similarly, for diseases, the fusion similarity calculation follows a parallel logic. The calculations are as follows:

5 $$\begin{aligned} SM= & {\left\{ \begin{array}{ll} \dfrac{MFS(m_i,m_j)+GFS(m_i,m_j)}{2},& {\text {if}}\ MFS(m_i,m_j)\ {\text {exists}}\\ {GFS(m_i,m_j),}& {\text {otherwise}} \end{array}\right. } \end{aligned}$$

6 $$\begin{aligned} SM= & {\left\{ \begin{array}{ll} \dfrac{DSS(d_i,d_j)+GDS(d_i,d_j)}{2},& {\text {if}}\ DSS(d_i,d_j)\ {\text {exists}}\\ {GDS(d_i,d_j),}& {\text {otherwise}} \end{array}\right. } \end{aligned}$$

**Fig. 1:**
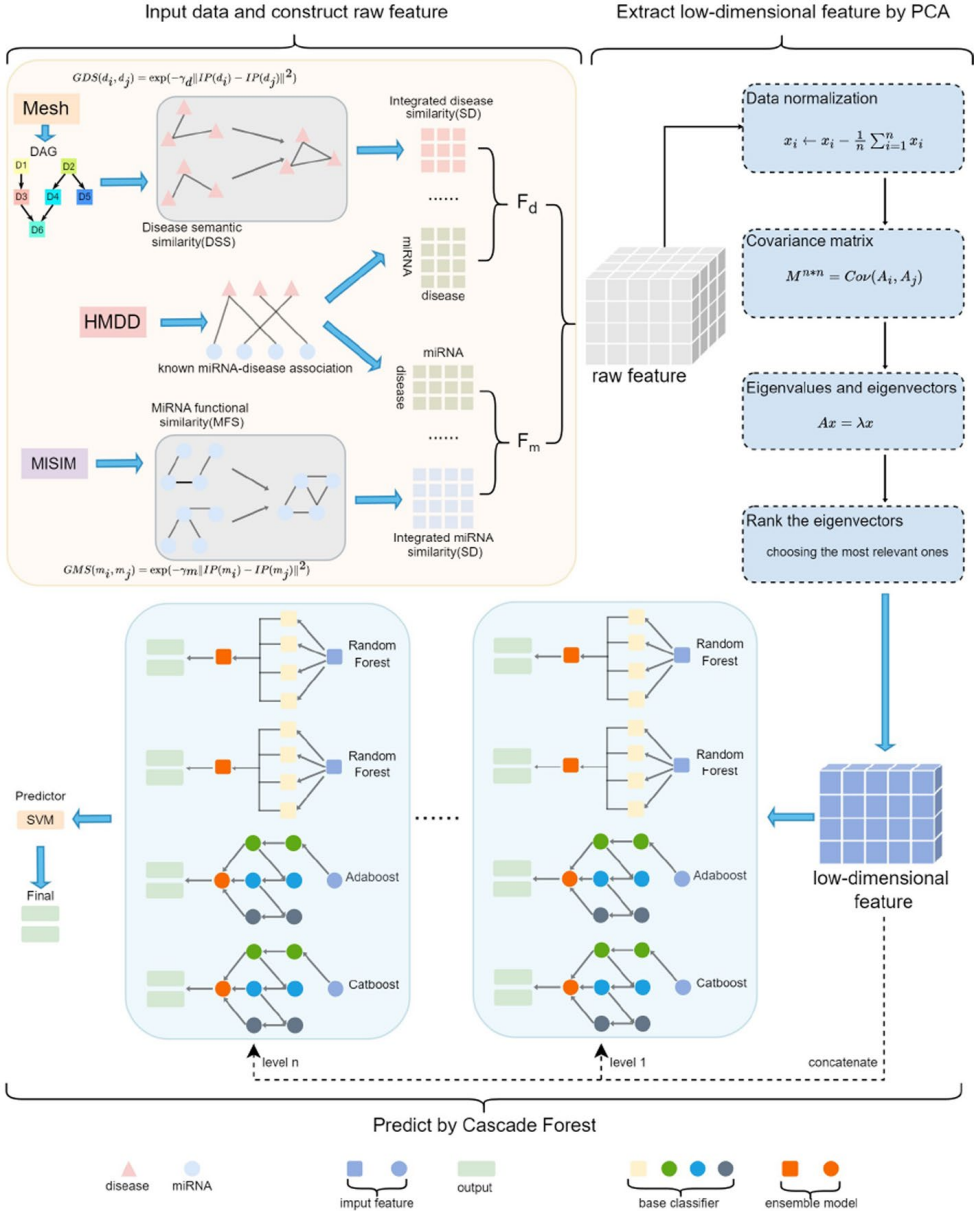
The structure of PCACFMDA

### PCACFMDA

This research introduces the PCACFMDA, a novel predictive framework, as illustrated in Fig. 1. Drawing inspiration from the study [38], we follow three steps to create high-quality feature representations. First, we form a 495-dimensional matrix ${SM}_{ij}$ by summarizing the fusion similarity between miRNAs, which quantifies the combined similarity between any two miRNAs in terms of function and interaction. Similarly, a 383-dimensional matrix, denoted as ${SD}_{ij}$, represents the integrated similarity among diseases, capturing the amalgamated disease similarities based on semantic attributes and interactions. Leveraging these matrices, we amplify the attributes of miRNAs and diseases. The resulting feature vectors encapsulate crucial information for each miRNA and disease, laying a robust groundwork for subsequent prediction of associations. The feature generation process can be elucidated through the following mathematical expressions:

7 $$\begin{aligned} F_m= & (SM_1A_1^{\prime },\cdots ,SM_1A_{383}^{\prime },\cdots ,SM_{495}A_1^{\prime },\cdots ,SM_{495}A_{383}^{\prime })^T \end{aligned}$$

8 $$\begin{aligned} F_d= & (SD_1A_1,\cdots ,SD_1A_{495},\cdots ,SD_{383}A_1,\cdots ,SD_{383}A_{495})^T \end{aligned}$$

where A contains 5430 corroborated association records and $A^\prime$ is its transposed form. The feature matrix $F_m$ was designed to contain 189,585 rows and 990 columns, with each row corresponding to a miRNA and each column representing a specific feature dimension. Similarly, the disease characterization matrix $F_d$ has 18,958 rows and 766 columns. To synthesize this multilevel information and facilitate the subsequent analysis, we perform an efficient horizontal splicing operation between $F_m$ and $F_d$ to generate a unified and comprehensive feature matrix F. This integrated feature matrix F not only contains information about the biological behaviors of miRNAs but also covers the complex associations between diseases, providing a strong basis for predicting the potential MDA. The formula is expressed as follows:

9 $$\begin{aligned} F=(F_m,F_d) \end{aligned}$$

where F denotes 189,585 samples and 1756 columns of characteristics. To maintain model training balance and accuracy, we chose cases with the same number of known positive associations from the remaining unconfirmed MDA as negative sample sets, totaling 10,860 samples.

By employing PCA, we achieve a concise feature representation through dimensional compression of relevant features. This approach eliminates extraneous variables while preserving the fundamental structure and patterns within the data, allowing the model to focus on learning the key features. This enhances prediction efficacy and reduces computational complexity. PCA transforms complex raw features into a combination of principal components via a linear transformation, with component weights established based on the data’s feature vectors. Notably, these principal components are ranked by their ability to explain data variance, thereby filtering out minor noise and redundant features. This process significantly contributes to advancing prediction model accuracy and resilience.

**Fig. 2:**
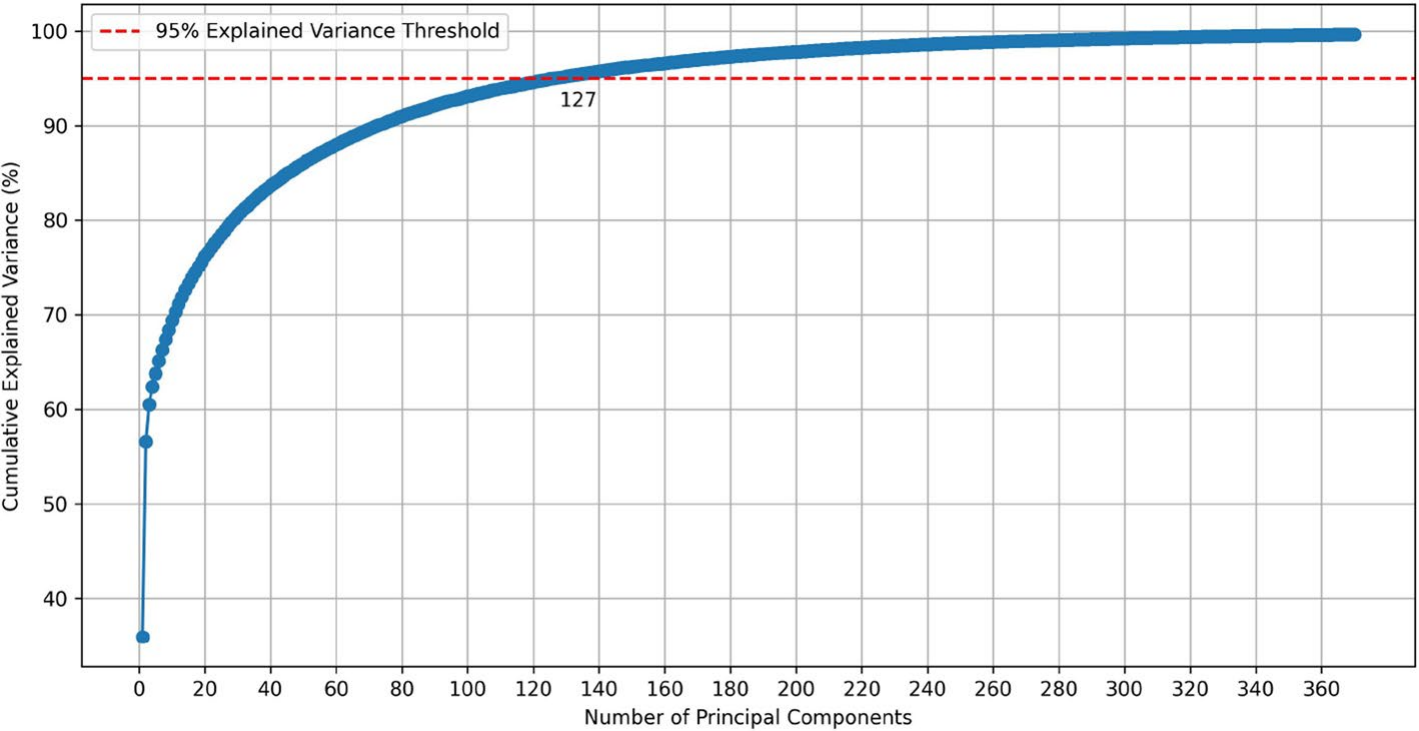
Cumulative explained variance by principal components

Specifically, we use the pre-constructed 1756-dimensional feature vectors as inputs to train the PCA model and extract the principal components. Next, we deeply analyze the percentage of variance explained by each principal component, to specify their respective shares of contribution to the overall data variability. By calculating and accumulating these variance percentages, we obtain a cumulative sequence of variance contributions, demonstrating how the degree of explanation of total data variability accumulates as the number of principal components increases. We analyzed the cumulative variance contribution of PCA, which was evaluated from 0% to 100% with an interval of 5%. The results show that the PCACFMDA model performs best when the cumulative variance contribution rate reaches 95%. Therefore, we chose 95% as the final cumulative variance contribution threshold. Our goal is to identify that key principal component ordinate that marks the first time the cumulative variance contribution exceeds the 95% threshold. Figure 2 visualizes this process of accumulating the variance contribution ratio, highlighting the balance between information retention and simplicity of the dimensionality reduction degree we chose, effectively promoting the double improvement of analysis performance and efficiency.

The Cascade forest, an efficient ensemble learning model, uses a series of multi-level random forests to gradually improve the accuracy. In this model, each level of random forest serves as the basic unit to specifically cope with the data samples that are not sufficiently differentiated from the previous level. Through this sequential refinement of the processing process, in-depth mining of data features and cumulative performance enhancement are realized. Compared to the complex deep neural network, cascade forest is superior in computational efficiency. It is highly interpretable and the process of tuning hyperparameters is easier.

**Fig. 3:**
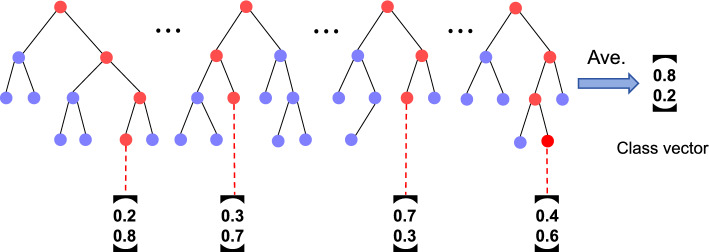
Decision-making processes in forest

This study revolutionizes the standard cascade forest architecture by constructing a composite model that integrates two random forests, an AdaBoost classifier and a CatBoost classifier. We evaluated the number of decision trees in these four classifiers ranging from 10 to 100 with an interval of 10. The results show that the PCACFMDA model performs best when the number of decision trees is 50. Therefore, we set the number of decision trees for all classifiers to 50. The integrated model has 200 decision trees distributed across four components, each with 50 trees, and tries to reduce reliance on computer resources while maintaining efficient computing speed via a highly optimized structure. Specifically, AdaBoost strengthens the learning of those samples that cannot be classified well by dynamically adjusting the sample weights and focusing on the samples misclassified by previous models. CatBoost uses advanced gradient boosting techniques and incorporates optimization strategies designed specifically for classification variables, as well as built-in regularization methods to improve model resilience.

Each forest within the cascade consists of multiple decision trees, with each tree producing a probability vector for a certain category. These individual tree outputs are aggregated and averaged across all trees in the forest to give the final decision result, as shown in Fig. 3. For this task, four independent predictors jointly contribute a set of eight probability values representing different confidence estimates for the two categories. These refined probability vectors, as additional features, are merged with the original data features to expand the feature space. The predictor for the cascade forest was set to SVM and assessed using linear, polynomial, and radial basis functions. The findings indicate that the PCACFMDA model performs most effectively with the polynomial kernel function. Consequently, the polynomial kernel function is selected as the SVM kernel function. To mitigate overfitting risks, a five-fold cross-validation approach is employed for all predictors during model training to enhance generalization capabilities. Notably, the model’s cascade structure is adaptable and not pre-set, operating as a dynamic strategy. Specifically, the model decides whether or not to extend its structure based on whether or not it can significantly improve the accuracy of cross-validation by adding new cascade levels. This adaptive mechanism allows the model to automatically optimize its complexity level based on the specific attributes and complexity of the data, eliminating the need to manually specify a fixed cascade depth.

### Experimental setup

After feature fusion, we obtained 189,585 samples with 1756 features, representing the integrated miRNA-disease association features. Following the undersampling process, we obtained 10,860 samples for model training. Subsequently, we used the PCA method from the sklearn library to determine the number of features that achieve a cumulative variance contribution ratio of 95%, thereby extracting low-dimensional features. For the cascade forest evaluator, we used 2 random forest classifiers, 1 AdaBoost classifier and 1 CatBoost classifier, each containing 50 decision trees. Meanwhile, the predictor used a support vector machine with polynomial kernel functions. The experiments were performed in a Windows 10 OS with a 12th Gen Intel Core i7-12700KF 3.60GHz CPU, an RTX 3090 Graphics card, and 64GB of RAM.

## Results

### Evaluation criteria

**Fig. 4:**
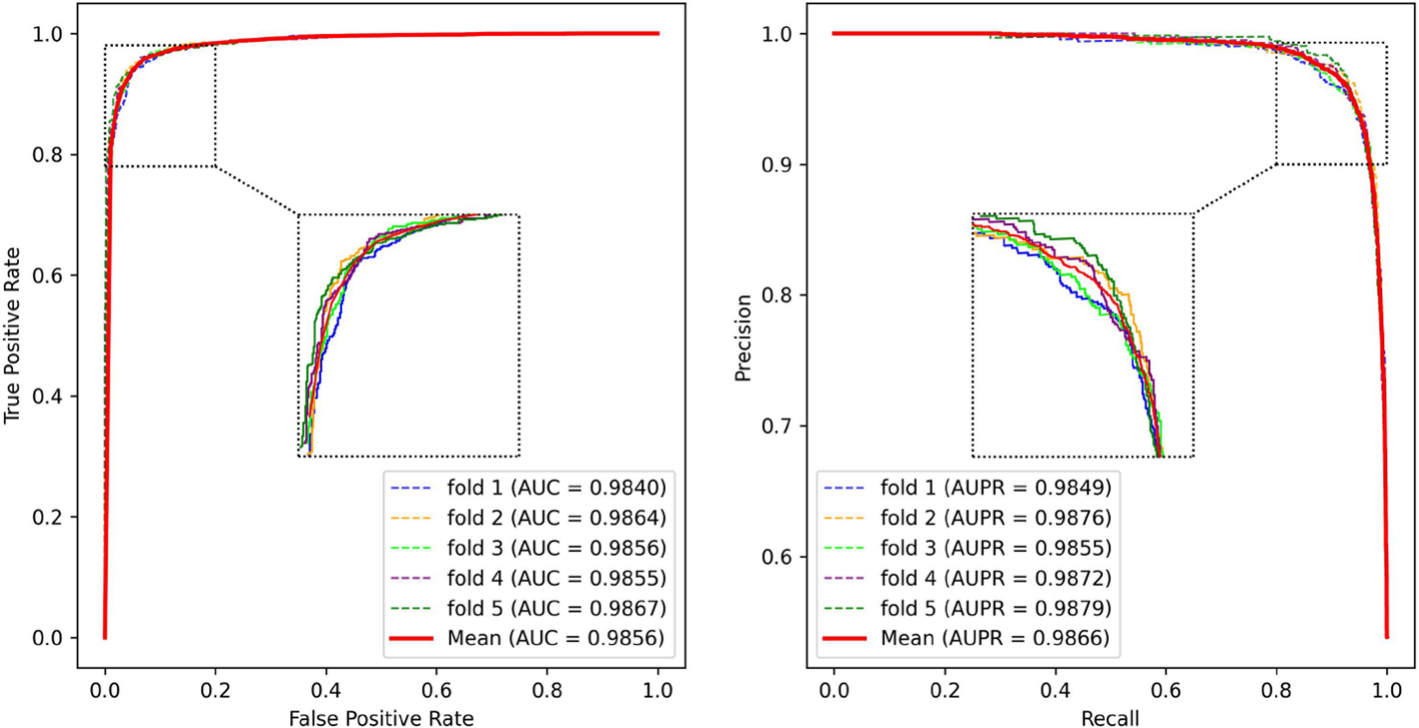
The ROC and PR curves of PCACFMDA in 5-fold cross-validation

**Fig. 5:**
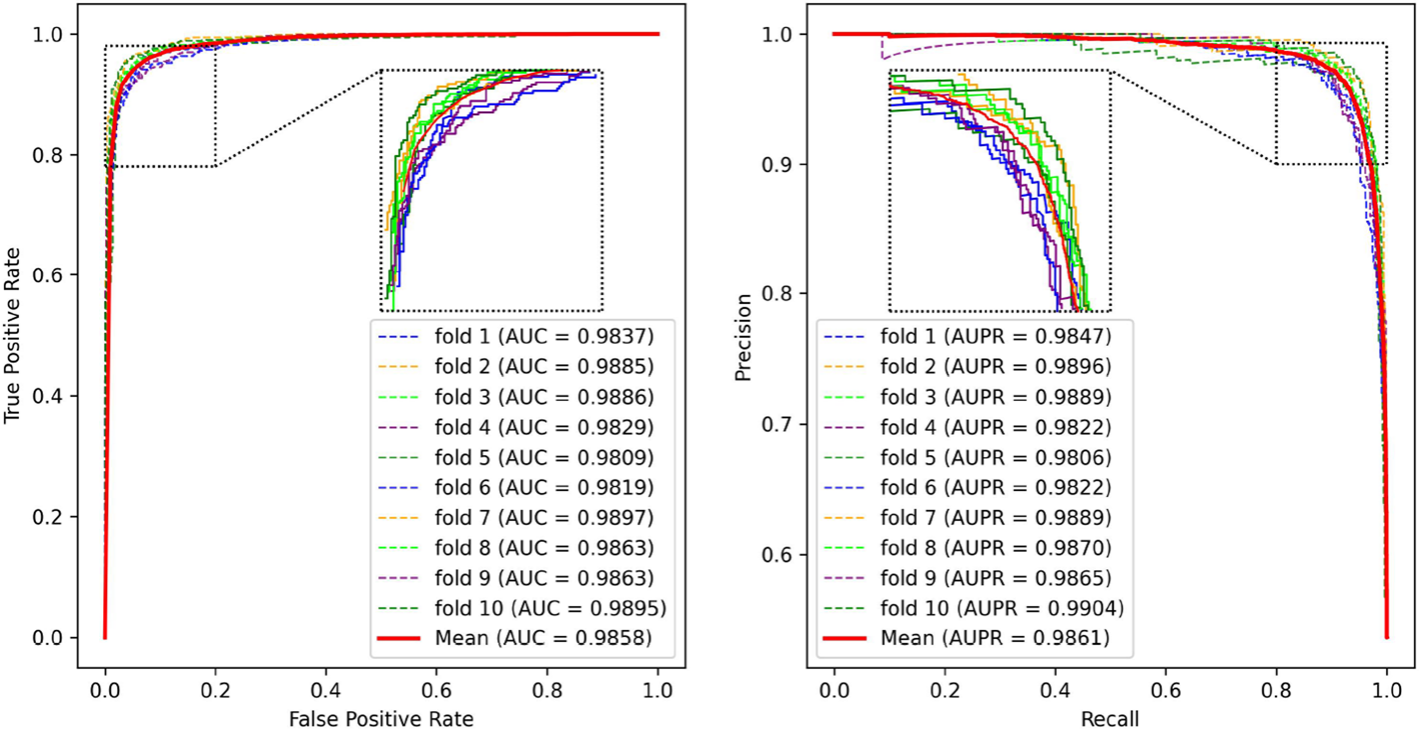
The ROC and PR curves of PCACFMDA in 10-fold cross-validation

To rigorously assess the efficacy of the PCACFMDA model, this study utilizes 5-fold and 10-fold cross-validation methods to enhance the reliability and robustness of the evaluations. Throughout the assessment, the discriminative power of the model is quantified by the area under the ROC curve (AUC), with higher AUC values signifying better predictive performance. For comprehensive performance analysis, a range of widely recognized evaluation metrics is employed, such as Accuracy (Acc), Precision (Pre), Recall (Rec), F1 Score (F1), and the area under the Precision-Recall Curve (AUPR). The calculations for Acc, Pre, Rec, and F1 are as follows:

10 $$\begin{aligned} Acc= & \frac{TP+TN}{TP+TN+FP+FN} \end{aligned}$$

11 $$\begin{aligned} Pre= & \frac{TP}{TP+FP} \end{aligned}$$

12 $$\begin{aligned} Rec= & \frac{TP}{TP+FN} \end{aligned}$$

13 $$\begin{aligned} F1= & \frac{2TP}{2TP+FP+FN} \end{aligned}$$

We initially performed a five-fold cross-validation on the PCACFMDA model. Figure 4 displays the outcomes, demonstrating an average AUC of 98.56%. The individual validation results were 98.40%, 98.64%, 98.56%, 98.55%, and 98.67%, respectively. Additionally, the model achieved an average AUPR of 98.66%, with individual validation outcomes of 98.49%, 98.76%, 98.55%, 98.72%, and 98.79%. The experimental results indicate that both the ROC and P-R curves confirm the performance and practicality of the PCACFMDA model.

Figure 5 depicts the outcomes of the PCACFMDA model after applying 10-fold cross-validation, achieving an average AUC of 98.58%. The result combines the specific scores obtained from the various rounds of validation, which are 98.37%, 98.85%, 98.86%, 98.29%, 98.09%, 98.19%, 98.97%, 98.63%, 98.63% 98.63%, and 98.95%. Of particular interest is the fact that the PCACFMDA model exhibits a subtle difference in the mean AUC values. These findings demonstrate the robustness and consistency of the algorithm. It can maintain a high and stable level of predictive efficacy across different sizes of training and testing set allocation scenarios.

### Ablation study

In this section, we constructed three models to explore the effectiveness of the proposed dimensionality reduction methods and the improvements made with the Cascade Forest approach. The descriptions of the models are as follows:

- Model A: This model combines a PCA dimensionality reduction method with an 80% variance contribution with an unmodified base cascade forest estimator.
- Model B: Compared to model A, model B employs a more stringent PCA setup, i.e., retaining the principal components with 95% variance contribution and continuing with the unmodified base cascade forest estimator.
- PCACFMDA model: This model adopts the same PCA dimensionality reduction strategy, retaining a 95% variance contribution rate. Simultaneously, the subsequent cascade forest estimator is fully optimized, enhancing both the diversity of the estimator and the accuracy of the predictor.

**Table 2:** Various performance indicators for Model A, Model B and PCACFMDA models

Methods	Acc (%)	Pre (%)	Rec (%)	F1 (%)	AUC (%)	AUPR (%)
A	88.08	87.80	88.47	88.12	95.15	94.95
B	89.57	89.56	89.60	89.57	95.78	95.90
PCACFMDA	94.48	94.74	94.18	94.46	98.56	98.66

**Table 3:** Various performance indicators for different classifiers

Methods	Acc (%)	Pre (%)	Rec (%)	F1 (%)	AUC (%)	AUPR (%)
SVM	86.84	86.84	86.83	86.84	94.29	94.11
LightGBM	88.28	87.64	89.14	88.38	95.12	94.84
RF	87.95	85.85	90.89	88.29	95.05	94.91
XGBoost	88.78	88.41	89.27	88.83	95.45	95.20
CF	89.31	89.66	88.89	89.27	95.64	95.70

All three models utilize the approach of averaging the prediction results to obtain the final prediction output, as illustrated in Table 2. PCA is employed for dimensionality reduction, while the cascade forest handles classification or regression tasks. The results demonstrate that increasing the number of principal components enhances the prediction performance of model B. Compared to model B, the PCACFMDA model exhibits superior performance across all evaluation metrics. Adjusting PCA parameters and optimizing the cascade forest estimator significantly improve the overall performance of the models.

### Comparative study of classifiers

**Fig. 6:**
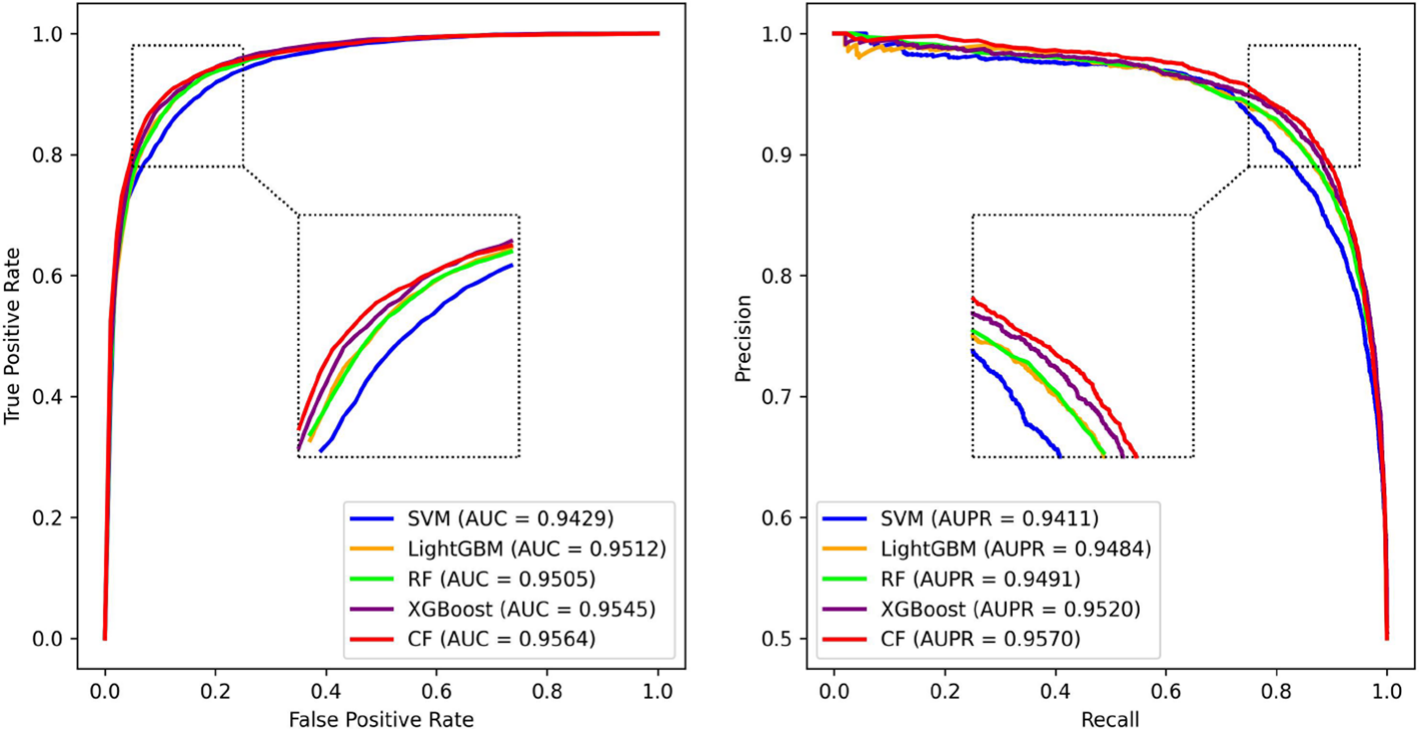
The ROC and PR curves of different classifiers in 5-fold cross-validation

In this section, we conducted experimental evaluations for the sample set. First, we obtained the feature data through PCA and applied various classifiers for comparative analysis, including SVM, LightGBM, Random Forest (RF), XGBoost, and Cascade Forest (CF). Among these, the number of decision trees was set to 100 for LightGBM, RF and XGBoost, while CF was configured with default parameters. The 5-fold cross-validation was performed on these classifiers.

The validation results are shown in Table 3 and Fig. 6. SVM performs relatively weakly in all evaluation metrics, especially in accuracy (86.84%), precision (86.84%) and F1 score (86.84%). LightGBM performs better in accuracy (88.28%), precision (87.64%), and F1 score (88.38%), but has better performance in AUC (95.12%) and AUPR (94.84%) are slightly inferior to CF. RF excels in recall (90.89%) but falls short of CF in other metrics, especially precision (85.85%) and F1 score (88.29%). XGBoost performs better in the aggregate, with accuracy (88.78%), precision (88.41%) and F1 score (88.83%) are close to CF, but slightly inferior to CF in AUC (95.45%) and AUPR (95.20%). CF demonstrates significant advantages in the five key evaluation dimensions, namely Acc, Pre, F1 Score, AUC, and AUPR. CF has an accuracy of 89.31%, a precision of 89.66%, an F1 Score of 89.27%, an AUC of 95.64%, and an AUPR of 95.70%, all of which are higher than the corresponding metrics of other classifiers. Although CF is not the absolute leader in recall, it excels in other core evaluation dimensions. Therefore, its potential and advantages in practical applications are further verified.

### Comparative study of models

In this section, we conduct a comparative analysis of the proposed PCACFMDA method against a selection of state-of-the-art prediction techniques, namely MDA-CF [37], ERMDA [60], DFELMDA [38], and CFSAEMDA [39]. Comprehensive explanations of each technique are provided below:

- The MDA-CF uses a cascade forest structure for MDA prediction, which internally combines two XGBoost models and two random forest models as base estimators.
- The ERMDA method employs a resampling strategy to generate multiple balanced small-scale training sets, trains independent learners on each subset, and applies a soft-voting mechanism to combine single learners’ prediction outcomes for their final choice.
- DFELMDA extracts low-dimensional expressions of miRNAs and disease features separately using a two-way deep AE, then gets correlation results for each type using deep random forests.
- CFSAEMDA employs a stacked AE to obtain the underlying feature representations and applies an improved cascade forest algorithm to accomplish the final prediction task.

**Fig. 7:**
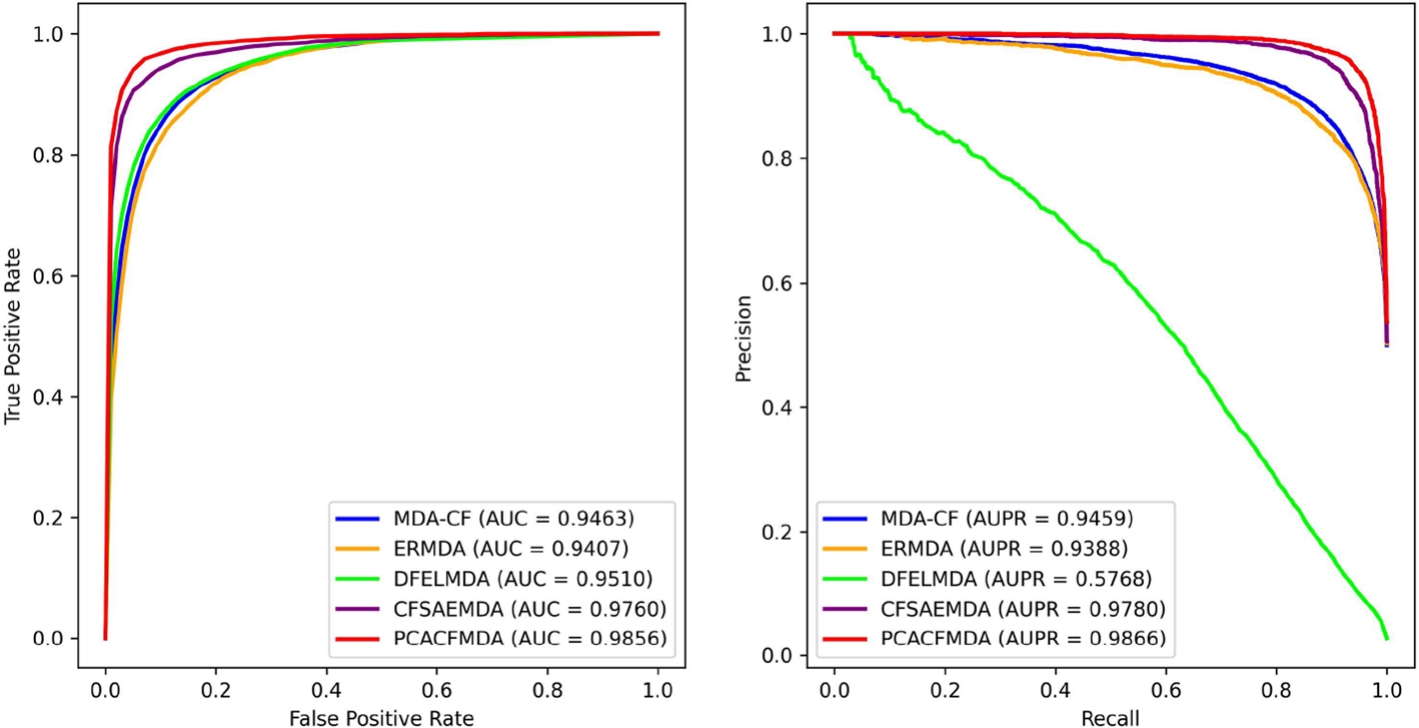
The ROC and PR curves of different models in 5-fold cross-validation

**Table 4:** Top 50 predicted miRNAs associated with BN, where H4, dbD and m2D represent HMDD v4.0, dbDEMC3.0 and miR2Disease

Rank	MiRNA	Evidence	Rank	MiRNA	Evidence
1	hsa-mir-29b	H4, dbD, m2D	26	hsa-mir-133a	H4, dbD
2	hsa-mir-345	H4, dbD	27	hsa-mir-31	H4, dbD, m2D
3	hsa-mir-199a	H4, dbD	28	hsa-mir-34c	H4, dbD
4	hsa-mir-21	H4, dbD, m2D	29	hsa-mir-19a	H4, dbD
5	hsa-let-7i	H4, dbD, m2D	30	hsa-let-7c	H4, dbD
6	hsa-mir-92a	H4, dbD	31	hsa-mir-429	H4, dbD, m2D
7	hsa-mir-125b	H4, dbD, m2D	32	hsa-let-7a	H4, dbD, m2D
8	hsa-mir-155	H4, dbD, m2D	33	hsa-mir-29c	H4, dbD, m2D
9	hsa-mir-214	H4, dbD	34	hsa-mir-205	H4, dbD, m2D
10	hsa-mir-1	H4, dbD	35	hsa-mir-200b	H4, dbD, m2D
11	hsa-mir-449b	H4, dbD	36	hsa-let-7g	dbD
12	hsa-mir-200a	H4, dbD, m2D	37	hsa-mir-192	H4, dbD
13	hsa-mir-34a	H4, dbD	38	hsa-mir-143	H4, dbD, m2D
14	hsa-mir-19b	H4, dbD	39	hsa-mir-198	H4, dbD
15	hsa-mir-146b	H4, dbD, m2D	40	hsa-mir-25	H4, dbD
16	hsa-let-7d	H4, dbD, m2D	41	hsa-mir-106b	H4, dbD
17	hsa-let-7e	H4, dbD	42	hsa-mir-9	H4, dbD, m2D
18	hsa-mir-29a	H4, dbD	43	hsa-let-7b	H4, dbD
19	hsa-mir-30a	dbD, m2D	44	hsa-mir-223	H4, dbD
20	hsa-mir-20a	H4, dbD, m2D	45	hsa-mir-26b	H4, dbD
21	hsa-mir-145	H4, dbD, m2D	46	hsa-mir-99b	H4, dbD
22	hsa-mir-125a	H4, dbD, m2D	47	hsa-mir-144	H4, dbD
23	hsa-mir-126	H4, dbD, m2D	48	hsa-mir-196b	H4, dbD
24	hsa-mir-221	H4, dbD, m2D	49	hsa-mir-7	H4, dbD, m2D
25	hsa-mir-195	H4, dbD, m2D	50	hsa-mir-96	H4, dbD, m2D

To ensure a fair comparison, the source codes of the MDA-CF, ERMDA, DFELMDA, and CFSAEMDA methods were appropriately adjusted to carry out this evaluation in the same experimental environment. Figure 7 shows the experimental results. In terms of AUC value, the PCACFMDA model achieves the highest at 98.56%. The CFSAEMDA and DFELMDA models are the next best, with 97.60% and 95.10%. The AUC values of the ERMDA and MDA-CF models are lower but still perform well. PCACFMDA performs well in AUPR assessment measures, with 98.66%. The CFSAEMDA model comes next with an AUPR value of 97.80%. The MDA-CF and ERMDA models also have high AUPR values of 94.59% and 93.88%. The DFELMDA model has a significantly lower AUPR value than the other models, which is only 57.68%. This set of data robustly confirms the effectiveness of the PCACFMDA method. Evaluated from both AUC and AUPR perspectives, PCACFMDA demonstrates superior predictive capability and higher accuracy.

**Table 5:** Top 50 predicted miRNAs associated with EN, where H4, dbD and m2D represent HMDD v4.0, dbDEMC3.0 and miR2Disease

Rank	MiRNA	Evidence	Rank	MiRNA	Evidence
1	hsa-mir-15a	H4, dbD	26	hsa-let-7i	H4, dbD
2	hsa-mir-150	H4, dbD	27	hsa-mir-10b	H4, dbD
3	hsa-mir-126	dbD	28	hsa-mir-140	H4, dbD
4	hsa-mir-146a	H4, dbD	29	hsa-let-7g	dbD
5	hsa-let-7a	dbD	30	hsa-mir-34a	H4, dbD
6	hsa-mir-203	H4, dbD, m2D	31	hsa-mir-18a	H4, dbD
7	hsa-mir-127	H4, dbD	32	hsa-mir-133a	H4, dbD
8	hsa-mir-103b	dbD	33	hsa-mir-106a	dbD
9	hsa-mir-21	H4, dbD, m2D	34	hsa-mir-199a	dbD
10	hsa-mir-155	H4, dbD	35	hsa-mir-146b	H4, dbD
11	hsa-mir-223	dbD, m2D	36	hsa-mir-193b	dbD
12	hsa-mir-19a	dbD	37	hsa-mir-30b	dbD
13	hsa-mir-221	dbD	38	hsa-mir-195	H4, dbD
14	hsa-mir-34b	H4, dbD	39	hsa-mir-198	dbD
15	hsa-mir-125a	H4, dbD	40	hsa-mir-483	H4, dbD
16	hsa-mir-16	H4, dbD	41	hsa-mir-29b	H4, dbD
17	hsa-mir-205	H4, dbD, m2D	42	hsa-mir-31	dbD
18	hsa-mir-27b	dbD	43	hsa-mir-27a	H4, dbD
19	hsa-mir-7	H4, dbD	44	hsa-mir-24	H4, dbD
20	hsa-mir-210	dbD	45	hsa-mir-124	H4, dbD
21	hsa-mir-200b	H4, dbD	46	hsa-mir-101	dbD
22	hsa-mir-200c	H4, dbD	47	hsa-mir-122	dbD
23	hsa-mir-148a	H4, dbD	48	hsa-let-7b	dbD
24	hsa-mir-20a	dbD	49	hsa-mir-141	dbD
25	hsa-mir-29a	dbD	50	hsa-let-7e	dbD

**Table 6:** Top 50 predicted miRNAs associated with LN, where H4, dbD and m2D represent HMDD v4.0, dbDEMC3.0 and miR2Disease

Rank	MiRNA	Evidence	Rank	MiRNA	Evidence
1	hsa-mir-17	H4, dbD, m2D	26	hsa-mir-145	H4, dbD, m2D
2	hsa-mir-146a	H4, dbD, m2D	27	hsa-mir-20a	H4, dbD, m2D
3	hsa-mir-133b	H4, dbD, m2D	28	hsa-mir-96	H4, dbD
4	hsa-mir-214	H4, dbD, m2D	29	hsa-mir-146b	dbD, m2D
5	hsa-mir-155	H4, dbD, m2D	30	hsa-let-7c	H4, dbD, m2D
6	hsa-mir-199a	H4, dbD, m2D	31	hsa-let-7f	dbD, m2D
7	hsa-mir-196a	H4, dbD	32	hsa-mir-31	H4, dbD, m2D
8	hsa-mir-29b	H4, dbD, m2D	33	hsa-mir-15a	H4, dbD
9	hsa-mir-34a	H4, dbD	34	hsa-mir-449b	dbD
10	hsa-mir-200b	H4, dbD, m2D	35	hsa-mir-93	H4, dbD, m2D
11	hsa-mir-27a	H4, dbD	36	hsa-mir-92a	H4, dbD
12	hsa-mir-148a	H4, dbD	37	hsa-mir-125a	H4, dbD, m2D
13	hsa-mir-152	H4, dbD	38	hsa-mir-205	H4, dbD, m2D
14	hsa-mir-195	H4, dbD, m2D	39	hsa-mir-125b	H4, dbD, m2D
15	hsa-mir-182	H4, dbD, m2D	40	hsa-mir-1	H4, dbD, m2D
16	hsa-mir-296	H4, dbD	41	hsa-mir-143	H4, dbD, m2D
17	hsa-mir-200c	H4, dbD, m2D	42	hsa-mir-16	H4, dbD, m2D
18	hsa-mir-126	H4, dbD, m2D	43	hsa-mir-132	H4, dbD
19	hsa-let-7a	H4, dbD, m2D	44	hsa-mir-130b	H4, dbD
20	hsa-mir-106b	H4, dbD	45	hsa-let-7b	H4, dbD, m2D
21	hsa-mir-30a	dbD, m2D	46	hsa-mir-29a	H4, dbD, m2D
22	hsa-mir-25	H4, dbD	47	hsa-mir-223	H4, dbD
23	hsa-mir-18a	dbD, m2D	48	hsa-mir-200a	H4, dbD, m2D
24	hsa-mir-21	H4, dbD, m2D	49	hsa-mir-141	H4, dbD, m2D
25	hsa-mir-183	H4, dbD, m2D	50	hsa-mir-30e	dbD, m2D

**Table 7:** Enrichment results for esophageal neoplasms-related miRNAs

KEGG pathway	*p*-value	miRNAs
Proteoglycans in cancer	4.31e−13	47
ECM-receptor interaction	6.00e−10	44
Hippo signaling pathway	7.31e−09	45
ErbB signaling pathway	2.27e−08	45
Fatty acid biosynthesis	9.19e−08	23
TGF-beta signaling pathway	9.19e−08	44
Pathways in cancer	6.28e−07	48
Rap1 signaling pathway	2.20e−06	47
Adherens junction	6.17e−06	44
Neurotrophin signaling pathway	8.02e−06	45
Renal cell carcinoma	1.23e−05	43
Focal adhesion	1.95e−05	47
MAPK signaling pathway	4.59e−05	47

**Fig. 8:**
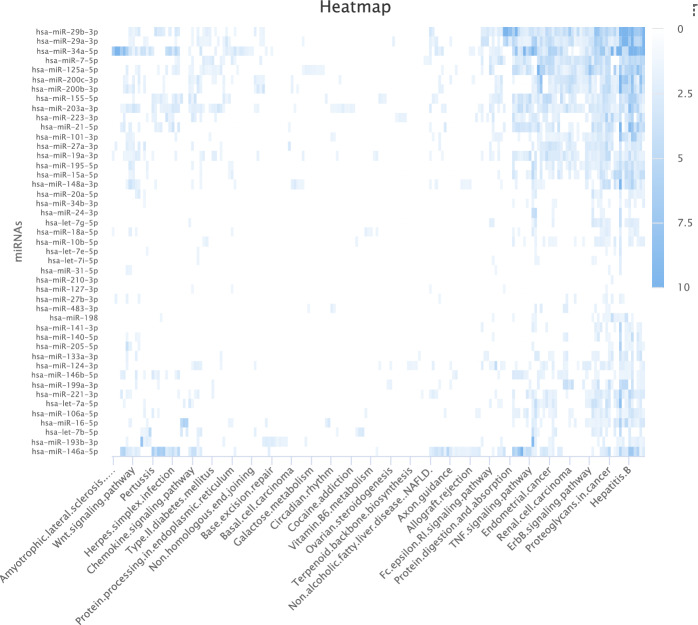
The illustration of heatmap based on esophageal neoplasms-related miRNAs

**Fig. 9:**
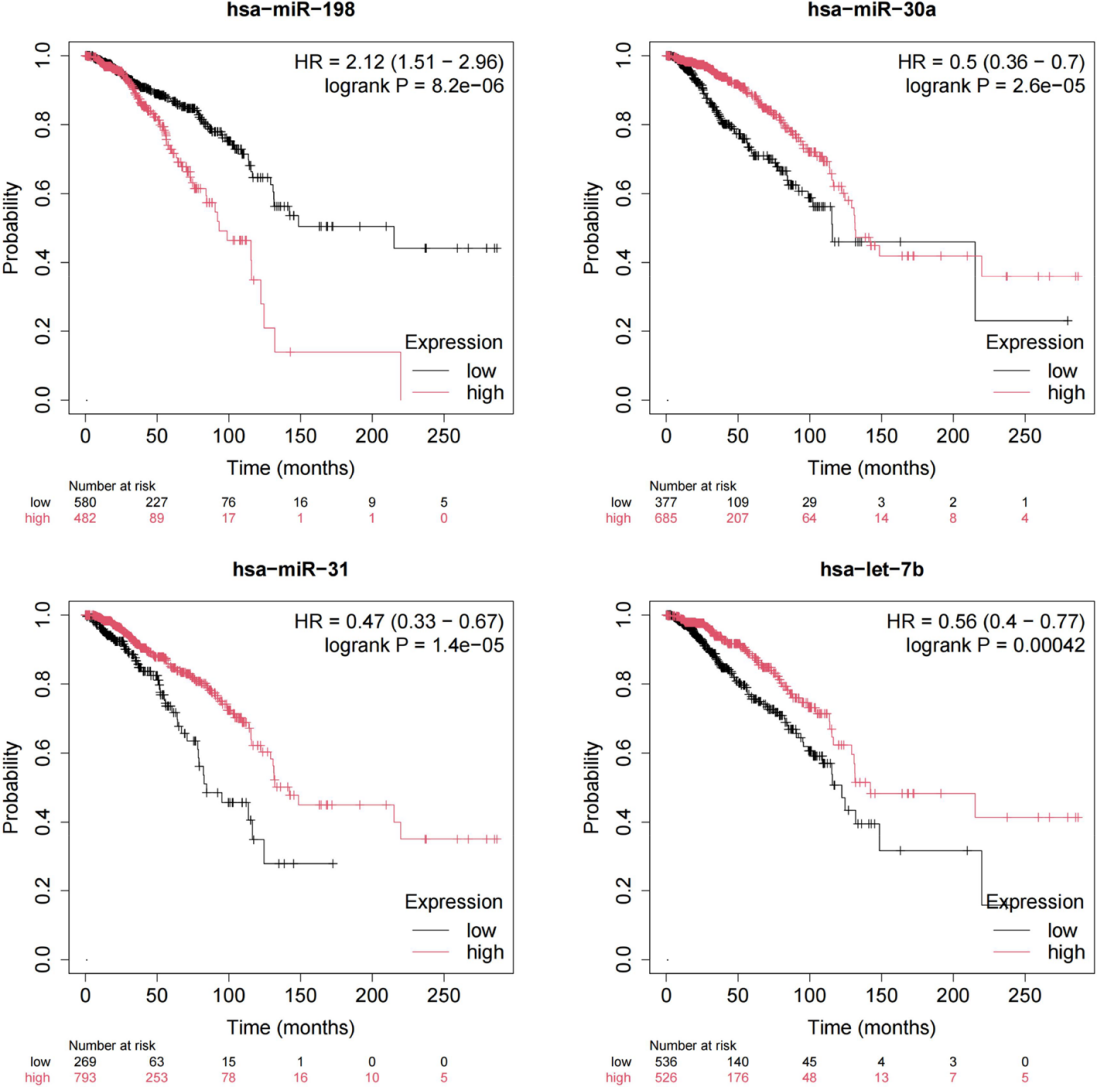
Kaplan-Meier plots of hsa-miR-198, hsa-miR-30a, hsa-miR-31, and hsa-let-7b for survival of patients with breast cancer

### Case study

To thoroughly substantiate the applicability and reliability of our proposed PCACFMDA model in real-world healthcare contexts, we meticulously plan and execute case studies focused on breast neoplasms(BN), esophageal neoplasms(EN), and lung neoplasms(LN). In particular, we start by removing from the dataset any known association records that are directly linked to a certain illness. Subsequently, the processed training set is adjusted using a balancing strategy to guarantee sufficient representation of all sample types. The PCACFMDA model then calculates probability scores of association to estimate the potential relationship between each miRNA and a specific disease. In the final step, based on the association probabilities determined by the model, we rank the miRNAs and select the top 50 corresponding miRNAs. The ranked lists are subsequently subjected to validation in three authoritative databases, dbDEMC3.0 [61], miR2Disease [62] and HMDD v4.0 [63].

BN is the most common cause of cancer-related mortality among women in industrialized countries. Although there have been tremendous advancements in medical therapies, metastatic and recurrent breast cancer are still a serious concern and present a significant barrier to clinical care. In light of this, it is important to explore novel strategies to deepen the understanding of breast cancer mechanisms. Notably, multiple studies have revealed that alterations in the expression of hsa-miR-29b, a tumor suppressor miRNA, may be an important biomarker indicative of recurrence and metastasis of the patient’s disease [64]. As shown in Table 4, the top 50 miRNAs most strongly associated with BN have been validated.

EN belongs to the category of highly prevalent neoplasms globally, in which benign neoplasms are mainly manifested as smooth muscle neoplasms. Squamous cell carcinoma, which ranks sixth in cancer-related mortality, is the most prevalent type of malignant neoplasm. Extensive scientific evidence indicates that dysregulated miRNA expression is intimately linked with the development of esophageal neoplasms. For instance, the down-regulation of hsa-mir-15a expression plays a crucial role in the genesis and progression of esophageal squamous cell carcinoma (ESCC). Therefore, the detection of serum levels of hsa-mir-15a is anticipated to serve as a novel diagnostic and prognostic biomarker with an important potential for clinical application [65]. As shown in Table 5, the top 50 miRNAs most strongly associated with EN have been validated.

LN involves aggregates of neoplasms originating in lung tissue or metastasizing to the lungs from other body parts, and metastatic conditions are specifically referred to as pulmonary metastatic neoplasms. Recent studies have shown a high correlation between miRNAs and the pathological processes involved in various lung cancers. The hsa-miR-133b [66] significantly inhibits cell proliferation in non-small cell lung cancer (NSCLC) by directly targeting the epidermal growth factor receptor (EGFR) and disrupting its downstream signaling pathways. This mechanism provides a revolutionary perspective for understanding and developing targeted therapies against EGFR-dependent cancers. Our prediction model selected the top 50 miRNAs associated with LN. As shown in Table 6, the top 50 miRNAs most strongly associated with LN have been validated.

### Pathway analysis

Using the DIANA-MirPath v.3 web tool [67], we analyzed the top 50 miRNAs in esophageal neoplasms to investigate miRNA-mediated pathway disruption and regulatory roles. As demonstrated in Table 7, pathways associated with esophageal neoplasms were substantially enriched, indicating that most candidate targets were closely related to those of esophageal neoplasms’ biological pathways. For example, reduced expression of the TGF-$\beta$ receptor in esophageal squamous carcinoma was associated with depth of infiltration, lymph node metastasis, pathological stage and poor prognosis [68]. ErbB family receptors, especially EGFR, play important roles in several cancer types. Studies have shown that mutations and overexpression of EGFR are also present in esophageal neoplasms [69] and are closely associated with disease progression. We also used mirPathDB 2.0 [70] to create a heat map of miRNA targets and associated pathways. As demonstrated in Fig. 8, deeper colors imply a greater association between miRNA targets and their respective pathways. In conclusion, the experimental results of PCACFMDA exhibited outstanding performance in predicting miRNA-disease correlations.

### Survival analysis

To assess the reliability of PCACFMDA, we employed miRpower [71] to statistically analyze breast cancer-related miRNAs. First, we used the TCGA database to identify miRNAs with p-values of less than 0.005, considered as prospective biomarkers for breast cancer diagnosis and prognosis. Then we focused on four specific potential miRNAs: hsa-miR-198, hsa-miR-30a, hsa-miR-31, and hsa-let-7b. To assess their potential impact on patient prognosis, we plotted Kaplan-Meier survival curves to visualize survival probabilities over time. If there is a significant difference between the survival curves of different groups, then these miRNAs could be the key factors influencing patient survival. As a result, we performed a detailed comparative analysis of the high-risk and low-risk breast cancer groups and showed that there was patients with some of the most prominent miRNA expression profiles had significantly different survival outcomes, highlighting the potential of these miRNAs as key biomarkers for prognostic and targeted therapies. By identifying and understanding these associations, we can better tailor treatment strategies to improve the prognosis of breast cancer patients. Figure 9 demonstrates that PCACFMDA efficiently extracts disease-related miRNAs and is a good technique for discovering prospective biomarkers in the biomedical area.

## Discussion and conclusion

MicroRNAs are key endogenous molecules that play crucial roles in post-transcriptional gene regulation, influencing numerous essential biological processes. In this study, we propose the PCACFMDA model to predict novel miRNA-disease associations. The model operates through three primary phases: First, we develop a framework that integrates multidimensional similarity features, capturing intricate patterns in the data. Next, PCA is applied to distill and refine deep structural information from these features. Finally, an enhanced cascade forest method is employed to predict potential miRNA-disease links accurately. Experimental results demonstrate that the PCACFMDA model not only achieves excellent predictive performance but also exhibits robustness and strong generalization capabilities. In future work, we aim to consider the impact of miRNA multi-targeting effects on disease association predictions. By utilizing additional bioinformatics tools and databases, such as TargetScan and miRTarBase, we plan to incorporate the influence of miRNAs targeting multiple mRNAs. Although our current model does not account for this factor, its performance on existing datasets validates its potential and reliability.

## Data Availability

The datasets that support the findings of this study are available in https://github.com/zhtdbobo/PCACFMDA.
